# Locating Sensors for Detecting Source-to-Target Patterns of Special Nuclear Material Smuggling: A Spatial Information Theoretic Approach

**DOI:** 10.3390/s100908070

**Published:** 2010-08-27

**Authors:** Jay Przybyla, Jeffrey Taylor, Xuesong Zhou

**Affiliations:** Department of Civil and Environmental Engineering, University of Utah, Salt Lake City, Utah, 84112, USA; E-Mails: jjpriz@gmail.com (J.P.); jeff.d.taylor@utah.edu (J.T.)

**Keywords:** Kalman filter, sensor network, nuclear material smuggling, information theory

## Abstract

In this paper, a spatial information-theoretic model is proposed to locate sensors for detecting source-to-target patterns of special nuclear material (SNM) smuggling. In order to ship the nuclear materials from a source location with SNM production to a target city, the smugglers must employ global and domestic logistics systems. This paper focuses on locating a limited set of fixed and mobile radiation sensors in a transportation network, with the intent to maximize the expected information gain and minimize the estimation error for the subsequent nuclear material detection stage. A Kalman filtering-based framework is adapted to assist the decision-maker in quantifying the network-wide information gain and SNM flow estimation accuracy.

## 1. Introduction

Many countries, including the United States, have determined that smuggling of Special Nuclear Material (SNM) for use as a weapon is a real threat to national security. The seriousness of this risk is apparent by the installation of SNM detectors in major international and domestic portals. Initially, the primary concern regarding the origin of SNM material was a result of the collapse of the Soviet Union and the accompanying economic depression that placed nuclear and radioactive materials into lower-security installations. However, the number of countries that have or may have gained access to SNM has grown recently and pose an ever-increasing risk of obtainment by those eager to smuggle it into possible target destinations and use it as a weapon. In order to ship the nuclear materials from a source location with SNM productions to a target city, the smugglers must employ the global and domestic transportation network in different modes, such as air cargo, container ships, and freight trucks. According to container port traffic statistics provided by the World Bank [1], there are about 470 million twenty-foot equivalent container units (TEU) shipped globally each year by these modes of travel, and 40 million containers enter the U.S. every year by land, sea, and air. This vast volume of containers is simply too large to practically and thoroughly search and screen.

Even with the existence of advanced network interdiction methodologies, the detection of SNM smuggling activities is very difficult. The smugglers can come from different countries, target different cities, and use many different routes and modes within the global transportation network. Improving system-wide observability of nuclear material smuggling flow in multimodal transportation networks, subject to available budgetary constraints, is an extremely challenging task that requires a seamless and complex integration of cyber and physical processes. The inability to quantify the information gain and system-wide impacts of individual detectors in a heterogeneous sensor network becomes a critical bottleneck in the evaluation of various promising detection scenarios.

### 1.1. Literature Review

#### 1.1.1. Network Interdiction Models

A number of optimization-based network interdiction models have been developed in the past few decades. In the well-known deterministic network interdiction model proposed by Wood [2], a smuggler attempts to maximize flow through a capacitated network while an interdictor tries to minimize this maximum flow by reducing flow on network arcs using limited resources. Wood’s study first proves that the network interdiction model is NP-hard, so it is computationally intensive even for solving its simplified form. By assuming a single smuggler or a group of fully coordinated smugglers, multiple potential sources are connected with a “super-source” and multiple targets are consolidated into a “super-sink”. Using a deterministic bi-level programming framework, Israeli and Wood [3] further considered a new objective function to maximize the shortest length (e.g., the least generalized cost) for the enemy (e.g., smuggler) to ship the material. In stochastic SNM network interdiction models proposed by Dimitrov *et al*. [4], the interdictor first installs radiation detectors on the network; the smuggler is assumed to know the locations of those sensors, and accordingly selects a route to avoid being detected. Using a stochastic optimization approach, their research aims to assist the interdictor in designing a robust sensor location plan that maximizes the possibility of detecting the smuggler.

All the above studies consider fixed sensors with models generally assuming a single source country (super source) and a single target country (super sink) with deterministic detection/interdicting rates. These simplifying assumptions are made because of the very difficult and unpredictable nature of the nuclear material smuggling network. There are likely sources and potential targets for SNM flow from which the research has constructed most likely source-to-target pairs. The above research is intended for general network interdiction problems; as such, the complicated error characteristics of SNM sensors and the use of mobile sensors have not been considered.

#### 1.1.2. Proposed Approach/Perspective

This paper will study the SNM detection and monitoring system based on an interdisciplinary approach, which represents a natural convergence of multiple fields including transportation engineering, nuclear engineering, and information theory. In fact, the transportation sensor network design problem has many similarities to the SNM detection problem. The comparison between the two networks is shown in Table 1.

The significant similarities include: (1) both systems are complex dynamic spatial systems organized around a hierarchical network structure; (2) both carry flows that vary dynamically, with varying degrees of predictability from origins to destinations; and (3) both have origin-to-destination pairs, and the traffic movement can be detected with point or point-to-point detectors.

The main differences between the two networks lie in their sensor detection probabilities, detection error distributions, levels of flow and the consequences of non-detection. Transportation networks often rely upon embedded inductive loop detectors and/or similar sensors which have high detection probabilities and produce very few false positives. These sensors are quite different from available nuclear material detection sensor systems, where operational complexity requires lower detection thresholds to improve detection probabilities, but consequently produces higher false alarm rates. This fundamental difference presents serious issues when propagating errors throughout the network and will be addressed in our future research. Additionally, transportation networks experience high flow volume, and errors in demand detection perpetuated by sensors have minimal consequences. SNM smuggling is a small probability event, and SNM is highly restricted and very difficult to obtain. The anticipated network flow of SNM is very low. However, errors in interdiction detection can have a significant impact with devastating consequences, resulting in high human and environmental casualties.

#### 1.1.3. Sensor Location Modeling

Due to the similarities between the transportation sensor location models used in transportation engineering for traffic origin-destination (OD) demand estimations and the SNM interdiction network, sensor location models for traffic OD demand estimation provides an excellent framework for the current research in detecting source-to-target SNM flow. Traffic OD demand information is a fundamental input for transportation network models to describe and predict spatially distributed traffic path/link flow patterns. For a typical metropolitan regional network such as San Francisco Bay Area, CA and Portland, OR, there are about 1,000 to 2,000 traffic analysis zones.

When the OD trip information desired is not easily obtained through surveys, transportation sensor networks are deployed to determine the traffic demand associated with a specific network. Lam and Lo [5] proposed “traffic flow volume” and “OD coverage” criteria to determine the priority of point detector locations. Yang *et al.* [6] presented a “maximum possible relative error (MPRE)” criterion to calculate the greatest possible deviation from an estimated demand table to the unknown true OD trip demand. Based on the trace of the *a posteriori* covariance matrix within a Kalman filtering framework, Zhou and List [7] proposed an information-theoretic framework for locating fixed sensors in the traffic OD demand estimation problem.

Information theory was developed by Claude Shannon [8] as a method to understand and solve problems with communication signals, and his concept of the measure of information has been successfully applied to many areas. The value of the information is measured by how additional information from sensor networks and other sources can help reduce or change the uncertainty involved with regard to a specific question. Quantifying the significance or value of a piece of information provides the ability to articulate and analyze the current state of a system and how any information changes might affect it. For example, Hintz [9] used a Shannon entropy-based model to locate sensors for tracking a single target moving in one dimension. Lee [10] proposed a conditional entropy-based framework to construct an atmospheric and geological sensor network design model. Recently, many studies, such as Zhao *et al.* [11] and Denzler and Brown [12], integrate information-theoretic measures into machine learning models and target detection models with mobile sensors.

In the area of mobile sensor network design, early studies (e.g., the survey paper Akyildiz *et al.* [13]) focused on how to improve the probabilistic spatial coverage of Base Stations (BS) in cellular phone networks. Recent research started to develop more rigorous optimization formulations. For example, Huang and Tseng [14] and Meguerdichian *et al.* [15] presented a maximal covering location model that aims to determine whether every point in the service area of the sensor network is covered by at least a fixed number of mobile detectors. In the above regular sensor network design applications, the unknown system states (e.g., the position and velocity of targets) typically can be directly measured by sensors. In comparison, sensing SNM flows in a transportation container network is difficult in its own right, as the nuclear signature identification and extraction problem involves complicated spatial mapping functions and a large number of unknown variables that are spatially and temporally correlated to one another.

More closely related to transportation networks, Neidhardt *et al.* [16], developed a Maximin Sensor Location Model (MSLM) to assist in detecting threats using “opportunistic” secondary mobile sensors to supplement information from stationary sensors. The mobile sensor location model utilizes sets of nodes identifying where monitoring is needed and where sensors can be placed to find the mobile sensor location configuration which maximizes the minimum coverage at nodes requiring monitoring. This methodology offers a probabilistic approach to determining potential network performance and cost, but cannot consider different sensor types or correlation between sensors.

### 1.2. Proposed Spatial Information Theoretic Approach

In spatial information theory, data is analyzed in reference to its spatial or topological characteristics. Often this is applied to transportation modeling where the flow of people, materials, or information goes through nodes in a complex three dimensional multi-modal network. This paper aims to apply a spatial information-theoretic framework to the problem of sensor placement in the SNM interdiction network, and to systematically assist the decision-maker in optimizing the network-wide information gain, system reliability, and total operating cost by deploying and networking fixed and mobile radiation sensors at different critical locations.

## 2. Problem Statement

Consider a transportation network with multiple origins *i* ∈ *I* (source cities) and destinations *j* ∈ *J* (target cities), as well as a set of nodes connected by a set of directed links. Given prior information on spatial source-to-target (ST) patterns in terms of smuggling SNM, the sensor location problem seeks to find a set of links *L'* so that smuggling detection and the detected SNM volume counts *c_l_* (e.g., the counts of detected nuclear smuggling activities) are available on link *l* ∈ *L'* for the subsequent smuggling ST flow estimation problem.

The goal of the sensor location problem is to maximize information gain from the sensor set on *L'*, subject to budget constraints for initial cost, installation, and maintenance. To obtain a significant information gain with respect to the prior ST flow estimate, focus must be placed on finding a detector matrix with adequate information. The information content of the detector matrix should provide a way to reduce the existing flow uncertainty. In this study, we assume the historical SNM smuggling ST flow information can be characterized by the *a priori* mean vector *D^−^* and the estimation error variance matrix *P^−^*.

### 2.1. Notation and Brief Definitions

The following notations are used to represent important components in the proposed sensor location model - namely the available measurements, estimation variables, mapping matrices between variables and measurements, and estimation error terms.

*m* = number of observations, or equivalent to the number of sensors if temporal factors are omitted,
*n* = number of ST pairs |*I*| × |*J*|,
*D* = ST flow vector, consisting of *n* elements *d*_(*i*,*j*)_, where *d*_(*i*,*j*)_ = ST volume with destination in zone *j*, originating their trips from zone *i*,
*D^−^* = *a priori* estimate of the mean values in the ST flow vector, consisting of *n* elements,
*D*^+^ = *a posteriori* estimate of the mean values in the ST flow vector,
*P^−^* = *a priori* error covariance matrix of ST flow estimate, consisting of (*n* × *n*) elements,
*P*^+^ = *a posteriori* error covariance matrix, *i.e.*, conditional covariance matrix of estimation errors after including measurements,
*H* = sensor matrix that maps unknown ST flows *D* to measurements *C*, consisting of (*m*×*n*) elements,
*p_(l)(i,j)_* = SNM link flow proportions, *i.e.*, proportion of smuggling ST flows from origin *i* to destination *j*, contributing to the passing flow on link *l*,
*K* = updating gain matrix, consisting of (*n* × *m*) elements,
C = SNM volume counts,
*R* = variance covariance matrix for combined errors, including measurement and modeling errors,
*ε* = combined error term, ε ∼ *N* (0, *R*).

The general SNM ST flow estimation process can be described through linear measurement equations in matrix form in Equation (1):

(1) C=HD+ε

or in functional form in Equation (2):

(2) $$c_{i}=\sum_{i,j}p_{\left(l)(i,j\right)}\times d_{\left(i,j\right)}+\omega_{l}$$

That is, the link flow count on link *l* is the sum of flow passing through link *l* from different source-to-target pairs plus a measurement error *ω_l_*

Measurement vector *C* typically includes link counts of SNM flow, and the size of *C* depends on the set of sensor locations. Sensor matrix *H* provides a linear mapping between ST flows and observations, and a typical example is a link flow proportion matrix that maps ST flows to link counts. In this study, we assume sensor matrix *H* can be determined from container flow assignment or simulation programs (within a standard traffic flow assignment framework), based on prior ST flow information. Additionally, we assume that the measurement error covariance matrix *R* is known. It should be noted that, although the actual values of sensor measurements are unknown before the sensors are installed, the analyst can estimate the magnitude of measurement errors from the same type of sensors at similar locations or related studies in other areas. In short, the given conditions for the sensor location problem can be mathematically summarized as: (1) the mean *D*^−^ and covariance matrix *P*^−^ of the *a priori* ST flow estimate and (2) the measurement error covariance matrix *R* and the sensor matrix *H* for all possible sensor sites.

### 2.2. Sensor Coverage Types

This study will consider both fixed and mobile sensors as part of a larger sensor network. Fixed sensors may represent a stationary SNM detection system like those found at ports of entry, while mobile sensors may represent hand-held or vehicle-mounted detection units. Both sensor types offer different levels of reliability and spatial coverage at various investment costs, and thus contain important factors to consider in sensor network design.

Mobile sensors are considered viable solutions when a large area, such as a seaport, an interstate highway system, or a metropolitan area, must be scanned in a short amount of time because they increase network coverage flexibility and introduce some degree of randomness to network coverage. On the other hand, mobile detectors typically have lower resolution and are more likely to be vulnerable to different types of noise or interference.

To overcome the inaccuracy and unreliability of a single mobile detector, a mobile sensor network (with multiple detectors) must work in a cooperative fashion to systematically improve its overall coverage [17]. Coverage randomness and flexibility is an important component in combating the effects of information asymmetry, which may arise in a situation where a smuggler knows the fixed sensor locations in a sensor network. Information asymmetry may lead to lower flow coverage due to smugglers trying to minimize their perceived detection risk. Random mobile sensor movement potentially decreases their ability to measure their risk exposure, thus limiting the value of this information. Furthermore, the benefit to using lower resolution but less expensive sensors is the ability to build large networks with fewer resources or increasing network coverage with the same resources. Issues relating to information asymmetry and cost-effectiveness will be addressed in our future research.

### 2.3. Objectives

In the following discussions, two practically critical and theoretically challenging issues will be addressed:

1. Fixed and mobile sensors with similar technologies might have correlated measurement errors. If the same sensor technology is placed throughout the network, then the correlation of the errors associated with the same technology will be high. A goal of this research is to understand the correlation between sensor technologies and locating and integrating correlated and uncorrelated, as well as fixed and mobile, sensors over an entire multi-modal transportation network to minimize the total detection errors and best interdict SNM smuggling flow.
2. The tradeoff between reliability and cost is fundamental to any technology-driven product. Simply purchasing the most costly and reliable sensors and placing them in a network does not necessarily produce the best results. A goal of this research is to understand the tradeoffs between equipment costs and detection accuracy and selecting the best combination of sensors and their locations to improve the system-wide reliability.

## 3. Kalman Filtering-Based Information Updating Process

**Figure 1.**
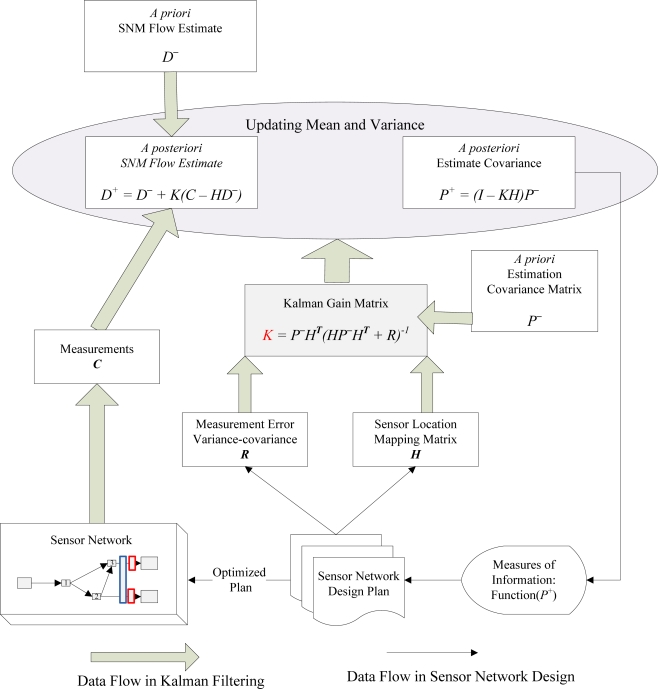
Data flow in Kalman filtering and sensor network design models.

This research uses Kalman filtering [18,19] to construct a spatial information-theoretic framework. As illustrated in Figure 1, the Kalman filtering model uses measurements *C* that are observed over time, which contain certain inaccuracies or random variations *R* (inherent to the type and coverage of sensors corresponding to detection matrix *H*), to predict values *D*^+^ that are closer to actual values.

The model accomplishes this by estimating the uncertainty of the predicted value *D^−^* and computing a weighted average of the predicted value and the measured value through the gain factor *K*. Kalman filtering has a distinct advantage over other information theory algorithms in that that current state estimates and covariances are put into matrices, which allows the algorithm to handle multiple dimensions in a single set of calculations.

In the proposed sensor network design model, the measure of information is calculated from the covariance *P*^+^, which is a measure of the estimated uncertainty of the system state estimate. This produces results that are closer to the actual values than either the estimate or measurements (with measurement error) produce alone. Finally, the scenario with the least uncertainty is assigned the highest importance to identify an optimized plan from a set of feasible sensor network design plans.

### 3.1. Step-by-Step Example

**Figure 2.**
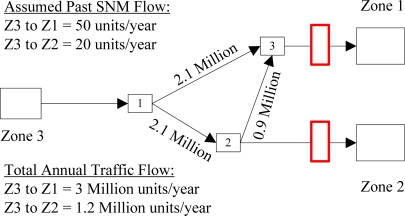
Example case to explain Kalman filtering.

For demonstrative purposes, let’s consider the following example: a transportation network with a single origin and two destinations (see Figure 2).

The single origin is a source city/area/region containing SNM and the two destinations are target cities with varying levels of attraction based on multiple factors related to smugglers’ malicious intent. In this example, Zone 1 has more attraction than Zone 2. The nodes within this network through which any flow can travel are labeled numerically 1, 2, and 3. The paths that can be traveled between Zone 1 and Zone 3 are {Z3, 1, 3, Z1} or {Z3, 1, 2, 3, Z1}. The only path to get to Zone 2 from the Zone 3 is {Z3, 1, 2, Z2}.

The different variables in the Kalman filter are explained in detail using the above example.

### 3.2. A Priori Estimates

*D^−^*—*a priori* state. This is a historical estimate as to the condition of the model before any filtering or prior to an update phase. These values can be estimated, based on historical data that is available, or synthesized from old sensors or counts. In the above example, we assume a historical flow of SNM material from Z3 to Z1 of 50 units/year and from Z3 to Z2 of 20 units/year. It should be noted that, the analysis time period could cover multiple years. Without loss of generality, one year is considered in our discussions. In comparison, we can assume the overall volume of container flow from Z3 to Z1 is 3 million units/year and the overall volume of flow from Z3 to Z2 is 1.2 million units/year, meaning that the SNM flow is only a very small faction of the overall flow to be scanned.

*P^−^*—*a priori* estimate covariance. This is an estimate of the condition of the current state. This does not include information from any additional sensors to be deployed. In this example, the variance of the SNM flow is estimated to be 4 for Z3 to Z1 and 1 for Z3 to Z2. The *P^−^* matrix for this example then would be $P^{-}=\left[\begin{array}{cc} 4 & 0\\ 0 & 1 \end{array}\right]$. These values correspond to a measurement error of ±2 for Z3 to Z1 and ±1 for Z3 to Z2. This matrix indicates there is no correlation between the variances on the two routes. Either the available data is accurate or the variance of SNM flow between the zones is mutually exclusive. If the diagonal values that are zeroes (0s) were either positive or negative numbers, the SNM flow variance would respectively be positively or negatively correlated, and, depending on the values, at least somewhat dependent on each other.

For instance, in the case of positive correlation for the two target areas, if the detected SNM flow to the first target area is significantly higher than expected, the flow to the second target area is likely to be higher than the forecast as well. Alternatively, as shown in Case 2, the sensor is unable to determine the destination of SNM flow because the detector is located at an origin ({Z3,1}). In this context, we might overestimate the flow to one zone and underestimate the flow to the other zone, which leads to a negative correlation between flow estimates to those two zones.

### 3.3. Updating Phase

In the updating phase, the prediction made in the predict phase *P^−^* is combined with the current state observation information to create a refined estimate of the current state. Some important variables that are used in the updating phase are:

*H*—Observation/sensor matrix. This variable is a matrix consisting of (*m x n*) elements that maps the sensor coverage of the model. The rows in the matrix represent the sensors in the network and the columns are the OD pairs. In the example, the *H* variable refers to the sensor coverage that is contained in the network. In this case, the red boxes in Figure 2 represent two sensors located on links {3, Z1} and {2, Z2}. This provides total coverage for the target cities with respect to any flow coming from Z3 on this network. The *H* matrix for this scenario would be $H=\left[\begin{array}{cc} 1 & 0\\ 0 & 1 \end{array}\right]$. The top row indicates that for sensor 1, the ST pair {Z3, Z1} is completely covered, but there is no coverage for ST pair {Z3, Z2}. The bottom row indicates that for sensor 2, the ST pair {Z3, Z1} is not covered, but there is complete coverage for ST pair {Z3, Z2}.

**Figure 3.**
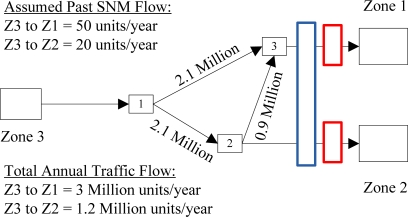
Example case to explain mobile sensor scenarios.

In the example presented in Figure 3, a mobile sensor is assigned to two network links, where the spatial coverage is represented by a long blue box. The mobile sensor is assumed to patrol each assigned link for an equal amount of time, which allows us to roughly estimate the flow covered by the sensor. For this scenario, the mobile sensor is an additional sensor to the network in Figure 2, thus an additional row is required for *H*. In this example, the mobile sensor spends 50 percent of its time on link {3, Z1}, where 100 percent of traffic between Z3 and Z1 flows, and 50 percent of its time on link {2, Z2}, where 100 percent of traffic between Z3 and Z2 flows. The *H* matrix for this example is $H=\left[\begin{array}{cc} 1 & 0\\ 0 & 1\\ 0.5 & 0.5 \end{array}\right]$, where the first two rows represent the fixed sensor coverage explained above, followed by the mobile sensor coverage in the third row.

**Figure 4.**
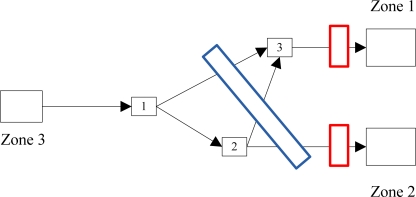
Example case to explain mobile sensor scenarios.

If the mobile sensor was assigned to cover links {1, 3}, {2, 3}, and {2, Z2}, as shown in Figure 4, the coverage would be very different. If we assume an equal time distribution between the three links (33 percent of total coverage time for each link) with random smuggler movement, the mobile sensor would cover one-third of the flow on link {1, 3}, one-third of the flow on link {2, 3}, and one-third of the flow on link {2, Z2}. Since 70 percent of the traffic between Z3 and Z1 occurs on link {1, 3}, the total coverage on link {1, 3} is 0.7 × 0.33 = 0.231. Similarly, the coverage on link {2, 3} is 0.3 × 0.33 = 0.099 and coverage on link {2, Z2} is 1 × 0.33 = 0.33. Total coverage for Z1 is the sum of the coverage for links {1, 3} and {2, 3}, which is 0.33, and total coverage for Z2 is 0.33. The H matrix for this scenario would be $H=\left[\begin{array}{cc} 1 & 0\\ 0 & 1\\ 0.231+0.099 & 0.33 \end{array}\right]=\left[\begin{array}{cc} 1 & 0\\ 0 & 1\\ 0.33 & 0.33 \end{array}\right]$. ε – Combined error term. This is a measurement of the error associated with the sensors in the system. The error term associated with the sensor will depend on the type of sensor. The error terms can also incorporate the unique nature of SNM sensors in that they have miss errors and false alarm errors. The error distribution for SNM sensors is often non-normal, introducing several issues when applied in the Kalman filter. Alternative approaches to the Kalman filter or data transformations may resolve these issues, which will be addressed in our future research. To illustrate an idealized case, the error rates for sensors in the examples provided are assumed to be simple values representing a normal error distribution for demonstration purposes only. To reflect the more realistic values in a future working model, actual rates will be determined from manufacturers or end users.

*R*—Variance covariance matrix for sensor counting errors, which include over-counting SNM volume (*i.e.*, false alarms) and undercounting SNM volume (*i.e.*, misses). Within the Kalman filter framework, it is important to understand system coverage as well as error correlation between sensors. If there is a large sensor error variance in the system, meaning the current sensors have significant noise, then there will be a small reduction in overall estimation uncertainty. Likewise, if the sensors have little noise, then this will result in a flow estimate with greater accuracy.

In the example in Figure 2, the *R* variable refers to the variance of the error associated with the two sensors that are located in the network. For this example, there is no correlation between the sensors which is reflected in the matrix by placing zeroes (0s) in the diagonals. If there were correlation between the sensors, then the zeroes (0s) in the matrix would be positive or negative numbers depending on the type of correlations. The R matrix for this scenario would be $R=\left[\begin{array}{cc} 1 & 0\\ 0 & 1 \end{array}\right]$.

*K*—Optimal Kalman gain. The goal of Kalman filtering is to minimize the variance of the estimation error. This is accomplished by minimizing the trace of the *a posteriori* estimate covariance *P^+^*. The matrix derivative is set equal to zero (Equation 3) and when solved for K, the equation for the optimal Kalman gain is obtained (Equation 4). This value is also known as the minimum mean square error (MMSE) estimate.

(3) $$\frac{\partial \mathrm{tr}(P^{+})}{\partial K}=-2{\left(HP^{-}\right)}^{T}+2K(HP^{-}H^{T}+R)=0$$

(4) $$K=P^{-}H^{T}{\left(HP^{-}H^{T}+R\right)}^{-1}$$

Using Equation (4), the K matrix for this scenario would be $K=\left[\begin{array}{cc} 0.8 & 0\\ 0 & 0.5 \end{array}\right]$.

The current state of the filter or update phase is represented by two variables:

*D^+^*—*a posteriori* state estimate. This is the condition of the filter after at least one round of the filter, representing what the system state will look like when combining the immediately previous state and the next estimated state. Kalman filtering is unique in that it does not need a long history of the state of the model to predict future states; it only relies on the immediately previous state and an estimate of the next state:

(5) $$D^{+}=D^{-}+K(C-HD^{\_})$$

In the example, if new SNM measurements obtained from the two fixed sensors in Figure 2 are 40 units (on link 3 to Z1) and 30 units (on link 2 to Z2), respectively, then we can update the estimate of the SNM flow count from Z3 to Z1 to 50 + 0.8 × (40 − 50) = 52, and the new estimate from Z3 to Z1 is increased to 20 + 0.5 × (30 − 20) = 25.

P^+^—*a posteriori* error covariance matrix. This is an estimated measure of the accuracy of the estimated state of the model. In other words, this could be considered an update of the current counting error. Understanding or measuring the amount of error associated with the estimates made during the filter is very important because it determines the weight assigned to each estimate in the model. The calculation of the *a posteriori* error covariance matrix is the heart of Kalman filtering and its derivation has essentially three steps. First, we can find a general update equation for P^+^:

(6) $$P^{+}=f(P^{-},K,H,R)$$

Because the optimal Kalman gain on minimizing *P^+^* is given in Equation (4), we can derive direct relation between *H* and *P^+^*:

(7) $$P^{+}=f(P^{-},H,R)$$

By definition, *P^+^* = *cov* (*D* − *D*^+^). As *D*^+^ can be derived from Equation (5) and C can be expressed from Equation (1), with some manipulation, we have:

(8) $$\begin{array}{ll} P^{+} & =\mathrm{cov}\left(D-(D^{-}+K(C-HD^{-}))\right)\\ & =\mathrm{cov}\left(D-(D^{-}+K(\mathrm{HD}+\varepsilon -HD^{-}))\right)\\ & =\mathrm{cov}\left((1-\mathrm{KH})(D-D^{-})-K\varepsilon \right)\\ & =\mathrm{cov}\left(\left(1-\mathrm{KH})(D-D^{-}\right)\right)+\mathrm{cov}(K\varepsilon )\\ & P^{+}=(1-KH)\mathrm{cov}(D-D^{-}){\left(1-\mathrm{KH}\right)}^{T}+K\mathrm{cov}(\varepsilon )K^{T} \end{array}$$

By considering the variance of measurement error as R, we have:

(9) $$P^{+}=(1-\mathrm{KH})P^{-}{\left(1-\mathrm{KH}\right)}^{T}+{\mathrm{KRK}}^{T}$$

A simplified format for optimal Kalman gain can be showed as follows:

(10) $$P^{+}=(I-\mathrm{KH})P^{-}$$

If the optimal gain factor is always to be derived from the Kalman filtering model, then the uncertainty format of the equation (without *K*) is:

(11) $$P^{+}={\left({\left(P^{-}\right)}^{-1}+H^{T}R^{-1}H\right)}^{-1}$$

The information format of the equation (without *K*) is given in Equation (12):

(12) $${\left(P^{+}\right)}^{-1}={\left(P^{-}\right)}^{-1}+H^{T}R^{-1}H$$

In the example given, and using Equations 9–12, it is possible to determine the *a posteriori* error covariance matrix when knowing the *a priori* error covariance matrix *P^−^*, the sensor coverage matrix *H*, and the variance covariance matrix *R*. Each of these terms has already been defined for the given example so the *P^+^* is easily calculated. For this example, the *P^+^* matrix would be $P^{+}=\left[\begin{array}{cc} 0.8 & 0\\ 0 & 0.5 \end{array}\right]$. The *P^+^* is the estimate of the new condition of the network after considering the current stage and the estimate of the next stage. The flow variance for each link is shown, and, when compared against the flow variance for the previous state (in this case, our baseline), we can see significant information gain. The variance on all links was reduced, indicating that there is better coverage than before. The sensor locations, types, and correlation can all be changed, depending on the scenario, to develop a model that creates the most realistic or idealistic coverage possible. The possibility exists that our information gain could be negative, indicating that the system coverage is worse than before. Either way, Kalman filtering provides an excellent solution to the problem of accurately understanding what and how much information gain is achieved between scenarios.

## 4. Measures of Information

One of the fundamental questions in both source-to-target SNM flow estimation and SNM sensor location problems is which criteria should be selected to drive the underlying optimization processes, (e.g., a measure of information or MOI). A measure of information in this study is considered a single value calculated from the *a posteriori* error covariance matrix *P*^+^ which is related to the variance in the estimated source-to-target SNM flow through the sensor network. By definition, the *a posteriori* error covariance matrix is:

(13) $$P^{+}=E\left\{[\tilde{D}-E(\tilde{D})]{\left[\tilde{D}-E(\tilde{D})\right]}^{T}\right\}$$

If the estimator is unbiased (*i.e*.,*E* (*D̃*) = 0), then the above equation reduces to:

(14) $$P^{+}=E(\tilde{D}{\tilde{D}}^{T})=E[(D-D^{+}){\left(D-D^{+}\right)}^{T}]$$

In the following, we first examine two commonly used estimation criteria, namely, the mean-square error and entropy, and then propose total flow variance as a new MOI to better consider estimation correlation.

### 4.1. Trace

The classic Kalman filter aims to minimize the mean-square error, that is, the Euclidean norm square of *D̃*:

(15) $$E{||\tilde{D}||}^{2}=E({\tilde{D}}^{T}\tilde{D})=E[{\left(D-D^{+}\right)}^{T}(D-D^{+})]$$

and is equal to the trace of the variance and covariance matrix:

(16) $$\mathrm{tr}(P^{+})=\mathrm{tr}(E[\tilde{D}{\tilde{D}}^{T}])$$

The trace of the variance covariance matrix is the sum of the diagonals of the matrix, which is equivalent to the total variance of a collection of uncorrelated random variables [20]. The trace can be calculated with Equation (17):

(17) $$\mathrm{tr}(P^{+})=\sum_{i=1}^{n}\mathrm{Cov}(X_{i},X_{i})=\sum_{i=1}^{n}\mathrm{Var}(X_{i})$$

Since the trace does not consider the effects of correlation between variables on the total variance, this measure of information does not consider the additional information which the covariance provides about the total flow. The covariance indicates some level of uncertainty in our error estimates, which helps to more accurately reflect the level of knowledge about the destination of the material moving within the network. By ignoring the covariance, the trace acts as a measure of information for the total system variance associated with different source-to-destination pairs. The trace measure is the preferred MOI when law enforcement agencies want to know precisely the SNM destination pattern in order to provide better proactive detection.

### 4.2. Entropy

Entropy is commonly used as a measure of information in information theory applications. For a discrete variable, Shannon’s original entropy [8] is defined as the number of ways in which the solution could have arisen. For a continuously distributed random vector *D*, on the other hand, the entropy is measured by *− E*(*ln f* (*D*)), where *f* is the joint density function for *D*. If *D* follows a normal distribution, then its entropy is quantified in Equation (18):

(18) $$\beta +\frac{1}{2}\ln(\det(P^{+}))$$

where *β* is a constant that depends on the size of *D*, that is, the number of source-to-destination SNM flow pairs in the context of SNM detection. The entropy measure is proportional to the log of the determinant of the covariance matrix. By ignoring the constant *β* and the monotonic logarithm function, we can simplify the entropy-based information measure for the *a posteriori* ST flow estimate as *det* (*P*^+^)

The determinant of the variance covariance matrix, as a measure of information, is also known as the generalized variance, which quantifies the uncertainty in our new estimate. Mathematically, the trace and determinant of the variance covariance matrix *P*^+^ can be calculated from the sum and product, respectively, of the eigenvalues of *P*^+^. Since the determinant considers the variance and covariance in the matrix, a smaller determinant is desirable because this indicates a more accurate estimate.

### 4.3. Total Flow Variance

This study proposes a Total Flow Variance measure, which considers the total variance of a weighted sum of SNM flow estimates. The total variance is calculated by Equation (19):

(19) $$\mathrm{Var}\left(\sum_{i=1}^{n}a_{i}X_{i}\right)=\sum_{i=1}^{n}a_{i}^{2}\mathrm{Var}(X_{i})+\sum_{i,j=1}^{n}a_{i}a_{j}\mathrm{Cov}(X_{i},X_{j})$$

where a_i_ and a_j_ can represent a constant defined as a “priority factor”. In other words, we can assign a value representing relative importance or priority to different targets and factor them into the total variance calculation during network design analysis so that sensor coverage is focused on high-value targets. By default, all the coefficients a_i_ is 1, so $\sum_{i=1}^{n}X_{i}$ indicates the total volume of SNM flow, and the total flow variance measure is equivalent to a squared estimation error for the total SNM flow.

The total flow variance includes two components: (1) the trace of the variance covariance matrix; and (2) the sum of the covariance for each pair of variables. As the detection accuracy to the total SNM volume (as opposed to individual source-to-target SNM flow and its spatial pattern) is the first priority in the early deployment of the sensor network, this MOI can capture the high-level and system-wide detection performance of a sensor network. In relation to the trace, the total flow variance can be viewed as a more appropriate measure for information about total flow coverage.

## 5. Discussion of Results

**Figure 5.**
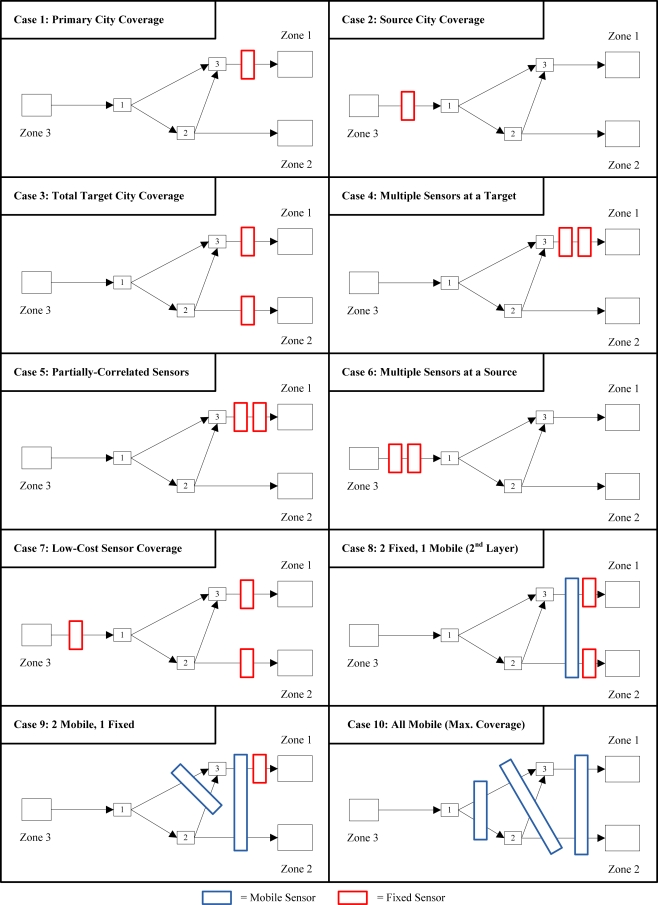
Illustrative examples for cases 1–10.

In Figure 5, we use illustrative examples (1–10) in the 6-node hypothetical transportation network introduced in Figure 3 to demonstrate how the proposed methodology can systematically evaluate the trade-offs between the accuracy, type, and placement of individual sensors for network-wide reliability. As identified in Section 3.1.1, $P^{-}=\left[\begin{array}{cc} 4 & 0\\ 0 & 1 \end{array}\right]$, with all other inputs and outputs identified in Table 2.

Compared to the general network interdiction models, this research explicitly recognizes the following inherent characteristics of SNM smuggling flow, as well as the coupling and interaction between the detection sensor network and the underlying multi-modal transportation network.

1. Correlated or uncorrelated measurement errors for networked detectors;
2. Probabilistic coverage of mobile or re-locatable sensors;
3. Different economic and risk impacts of miss rate and false alarm rate; and
4. Impact of spatial source-to-target SNM smuggling flow distribution on sensor network design.

### 5.1. Fixed Sensor Cases

Of the 10 illustrative examples in Figure 5, Cases 1–7 consider deployment of fixed sensors only. Cases 1, 4, and 5 use fixed sensors that are focused on protecting Zone 1 with a higher priority. Case (3) uses fixed sensors that cover both target cities. Cases 2 and 6 focus on covering flow from source Zone 3. Case 4 uses two sensors with partially correlated errors at the same location. Case 7 considers three inexpensive SNM detectors with relatively large levels of measurement error equally distributed throughout the network to cover both targets and the source. By comparing the associated trace values of different cases, which are the total measurement errors, we have the following insightful remarks:

Case 1 is better than Case 2, as the sensor location closer to the target provides more information about SNM flow destination. Both Cases 4 and 5 fully cover the smuggling flow to Zone 1, but it is more desirable to deploy two uncorrelated sensors to reduce total measurement errors. Case 3, which fully covers both target cities, reduces the overall system risk compared to Cases 1 and 4. If the total cost of the three sensors in Case 7 is lower than the cost of the two expensive and accurate sensors in Case 3, then Case 7 is actually a more cost-efficient and reliable option with increased system redundancy.

### 5.2. Mobile Sensor Cases

Cases 8–10 represent various levels of mobile sensor implementation. Using Case 3 as a baseline for comparison, where the two target zones are each covered by a fixed sensor, the network is manipulated by either adding mobile sensors or replacing a fixed sensor with a mobile sensor. Case 8 allows us to briefly examine the merits of using mobile sensors for improved overall network coverage. Case 9 examines a situation in which mobile sensors are used primarily to improve coverage for Zone 1, which may be similar to a scenario in which mobile sensors are assigned to protect specific targets due to their perceived priority or vulnerability. Case 10 considers a network composed entirely of low-resolution mobile sensors to increase stochastic network coverage.

In Case 8, a mobile sensor is added to the network, which covers 50 percent of flow to Zone 1 and 50 percent of flow to Zone 2. Comparing the results shown in Table 2, Case 8 shows a small reduction in the trace and total flow variance over the base Case 3. This indicates that network coverage and knowledge about possible destinations for SNM has increased under the new network design. Case 8 may also be compared to Case 7, where the lower total flow variance in Case 7 indicates greater network coverage than in Case 8. However, the sensor location near the source in Case 7 limits our knowledge about the destination of the SNM, which is revealed by a slightly higher trace in Case 7 than in Case 8. These results indicate that a fixed sensor scanning traffic leaving a source has a greater level of uncertainty regarding the destination, while the less accurate mobile sensor scanning near the destination delivers a higher level of certainty about the destination, which may have greater utility for organizations responding to specific national security threats

In terms of overall network coverage objectives, Case 9 is similar to Case 1 and Case 4—both are focused on improving coverage for Zone 1. The coverage conditions in Case 9 assign three sensors to the paths leading to Zone 1, which includes one fixed sensor on link {3, Z1}, one mobile sensor assigned to cover 50 percent of the total flow on links {1, 3} and {2, 3}, and another mobile sensor which covers 50 percent of the total flow to Zone 1 and 50 percent of the total flow to Zone 2.

The results in Table 2 show a modest information gain in Case 9 compared to Case 1. The improvement in the trace is less than the improvement in the total flow variance, which indicates that the additional mobile sensors have provided more information about total network flow than flow destination.

When we compare Case 9 to Case 4, the presence of the mobile sensor partially covering the path to Zone 2 obviously provides better coverage for that link. In addition to Zone 2 receiving coverage, the entire network coverage is increased as seen by the lower total variance. However, Case 4 has a lower trace value than Case 9. By focusing on covering Zone 1 with two fixed sensors, Case 4 provides more information about SNM destination due to the presence of the uncorrelated, accurate sensor on the same higher priority link. This situation creates a unique perspective in tradeoffs. Multi-layered coverage with a greater number of less accurate mobile sensors was potentially less effective compared to multi-layered fixed sensors because they provided less information about SNM destination, but the results also show the benefits of accurate flow estimates for tailoring coverage to higher-priority targets. We must also consider that total coverage and destination uncertainty for mobile sensors is affected by differences in traffic flow on the links covered by the mobile sensor and its accuracy. This framework can be used to investigate the cost-benefit relationship between sensor types in different coverage scenarios as well as the tradeoff these different scenarios present with regard to accurate flow estimation.

Case 10 is a special situation in which we try to maximize the network’s stochastic coverage with multi-layered mobile sensors assigned without regard for previous network information or conditions. Mobile sensors are used to cover every link between Node 1 and Zones 1 and 2, with overlapping coverage to offer the ability to cover both zones with at least one sensor at any given time. Immediately we notice a relatively poor information gain, especially when compared Case 7, which identifies the limited utility of employing mobile sensors compared to fixed sensors.

Case 10 may be interpreted as a situation in which mobile sensors replace the fixed sensors in Case 7, either for improved network flexibility, lower cost, perceived benefits associated with greater geographical coverage, and/or more random coverage. These results further highlight the potential benefits for utilizing this framework for intelligent sensor network design.

## 6. Sensor Network Design for a Large-scale Network

The proposed sensor network design model is essentially a special case of the discrete network design problem [21], so an integer programming model can be constructed to find the optimal sensor location solution through a branch-and-bound search tree. Each node in the search tree represents a decision to install an additional fixed or mobile nuclear material radiation sensor on a single or a set of transportation locations. To reduce the number of nodes to be computed, we use a beam search heuristic algorithm, which branches from the nodes level by level but only keeps a fixed number of promising nodes (in terms of a certain measure of information) at each level. Essentially, the total computational time of the proposed beam search algorithm is determined by the number of nodes to be evaluated.

To apply the proposed theoretical framework to a realistic transportation network, we can use the following procedure (with a nation-wide railroad network as an example):

- Construct a transportation network representation with nodes and links. For example, the U.S. railroad network has 10,560 nodes and 25,882 links.
- Setup an analysis zonal structure: Entry ports represent origins, hub and classification yards represent potential sensor locations, and large metropolitan areas represent potential destinations.
- With inputs from DHS or other agencies, setup a prior source-to-target destination matrix, and estimate the uncertainty range of each source-to-target pair to construct *P*^−^.
- Perform traffic assignment process and assign commodity flow to different paths according to the existing routing plans of railroad companies.
- Since SNM flow is an extremely small fraction of total commodity flow, we need to also estimate the screening coverage associated with different detection technologies and policies.
- Finally, generate the H matrix based on the above four factors: (1) underlying network, (2) OD pattern for column, (3) source-to-target pair-link incidence matrix (each link is a row), and (4) percentage to be scanned.
- Obtain *R* matrix using estimates of sensor error.

In our preliminary computational experiments, for a large-scale transportation network with about 1,000 possible source-to-target pairs, evaluating the measures of information at each search node typically takes less than one second on a regular personal computer. The application of this method to the actual SNM sensor network would take cooperation with the agencies involved in placement of the sensors. The extent to which the SNM sensor network is cataloged is unknown and may take extensive effort to model.

## 7. Conclusions

This paper has presented a new information-theoretic methodology, based upon Kalman filtering, for analyzing and designing a sensor network to better detect international, regional, and local SNM flow. Our approach utilizes an existing transportation network with origins and destinations with varying levels of detail (e.g., city-to-city, country-to-city) and potential paths between them (e.g., land, sea, and air), to determine the information gain from different sensor network configurations. The results render a predicted level of protection for specific destinations in the network and allow policy makers to experiment with different sensor deployment strategies. Options, such as deploying more mobile sensors instead of fixed sensors or analyzing the cost effectiveness of fully screening cargo containers for detecting smuggled nuclear threats, can be explored and analyzed mathematically. The results of our decision making support framework may allow policy makers to better evaluate specific strategies employed to detect SNM movement. For example, one might consider the benefits of installing mobile or fixed sensors in or near source zones to detect nuclear material smuggling, compared to the benefits of deploying high-density sensor networks in target areas. Ultimately, contributions from this framework may lead to significant reductions in uncontrolled SNM traffic, with broad implications for preventing nuclear proliferation and nuclear terrorism, which are widely acknowledged as fundamental threats to global security and stability.

At this time, this framework does not consider cases of asymmetric information (e.g., smuggler knows sensor locations), traffic patterns, or missed detection time while moving between the assigned links. These factors are significant concerns when designing and implementing real-world sensor networks and thus will be incorporated into future research to further enhance its analytical capabilities. In addition, new radiation sensing technologies [22] and research results from the fields of nuclear forensic science [23] should be also incorporated into our proposed model to better trace the origin of illicitly trafficked nuclear materials.

## Figures and Tables

**Table 1.** Comparison of Transportation and SNM Smuggling Networks.

	**Transportation (Passenger and Freight) Flow**	**Nuclear Material Smuggling Flow**
Origin	Home/office/warehouse	Source city
Destination	Warehouse/office/home	Target city
Detectors	In-pavement loop detectors, road-side vehicle counting stations, vehicle identification readers	Fixed and handheld detectors, Radio Frequency Identification (RFID) detector
Flow Volume	High	Extremely low
Objective	Improve system observability to maximize system mobility	Improve system observability to maximize SNM flow to be interdicted

**Table 2.** Example descriptions, intermediate steps, and calculation results.

**Case**	**Description**	**Inputs/Outputs**	**Trace**	**Det.**	**Entropy**	**Total Flow Var.**
1	One fixed sensor covers Zone 1	$H=[\begin{array}{cc} 1 & 0 \end{array}]\;\;\;R=1\;\;\;P^{+}=\left[\begin{array}{cc} 0.8 & 0\\ 0 & 1 \end{array}\right]$	1.800	0.800	−0.223	1.800
2	One fixed sensor covers Zone 3	$H=[\begin{array}{cc} 1 & 1 \end{array}]\;\;\;R=1\;\;\;P^{+}=\left[\begin{array}{cc} 1.33 & -0.67\\ -0.67 & 0.83 \end{array}\right]$	2.167	0.667	−0.405	0.833
3	Total coverage for Zones 1 & 2 (one fixed sensor each)	$H=\left[\begin{array}{cc} 1 & 0\\ 0 & 1 \end{array}\right]\;\;\;R=\left[\begin{array}{cc} 1 & 0\\ 0 & 1 \end{array}\right]\;\;\;P^{+}=\left[\begin{array}{cc} 0.8 & 0\\ 0 & 0.5 \end{array}\right]$	1.300	0.400	−0.916	1.300
4	Two fixed sensors cover Zone 1	$H=\left[\begin{array}{cc} 1 & 0\\ 1 & 0 \end{array}\right]\;\;\;R=\left[\begin{array}{cc} 1 & 0\\ 0 & 1 \end{array}\right]\;\;\;P^{+}=\left[\begin{array}{cc} 0.44 & 0\\ 0 & 1 \end{array}\right]$	1.444	0.444	−0.811	1.444
5	Two partially-correlated fixed sensors cover Zone 1	$H=\left[\begin{array}{cc} 1 & 0\\ 1 & 0 \end{array}\right]\;\;\;R=\left[\begin{array}{cc} 1 & 0.25\\ 0.25 & 1 \end{array}\right]\;\;\;P^{+}=\left[\begin{array}{cc} 0.54 & 0\\ 0 & 1 \end{array}\right]$	1.541	0.541	−0.615	1.541
6	Two fixed sensors cover Zone 3	$H=\left[\begin{array}{cc} 1 & 1\\ 1 & 1 \end{array}\right]\;\;\;R=\left[\begin{array}{cc} 1 & 0\\ 0 & 1 \end{array}\right]\;\;\;P^{+}=\left[\begin{array}{cc} 1.09 & -0.73\\ -0.73 & 0.82 \end{array}\right]$	1.909	0.364	−1.012	0.455
7	Three low-cost fixed sensors cover Zones 1, 2, and 3.	$H=\left[\begin{array}{ll} 1 & 0\\ 0 & 1\\ 1 & 1 \end{array}\right]\;\;\;R=\left[\begin{array}{ccc} 1.5 & 0 & 0\\ 0 & 1.5 & 0\\ 0 & 0 & 1.5 \end{array}\right]\;\;\;P^{+}=\left[\begin{array}{cc} 0.72 & -0.21\\ -0.21 & 0.49 \end{array}\right]$	1.205	0.308	−1.179	0.795
8	Two fixed sensors: #1 covers Zone 1, #2 covers Zone 2. One mobile sensor covers half flow to both Zone 1 and Zone 2.	$H=\left[\begin{array}{cc} 1 & 0\\ 0 & 1\\ 0.5 & 0.5 \end{array}\right]\;\;\;R=\left[\begin{array}{ccc} 1 & 0 & 0\\ 0 & 1 & 0\\ 0 & 0 & 1.5 \end{array}\right]\;\;\;P^{+}=\left[\begin{array}{cc} 0.71 & -0.05\\ -0.05 & 0.47 \end{array}\right]$	1.178	0.329	−1.112	1.068
9	Two mobile sensors: #1 covers both paths to Zone 1, #2 covers both Zone 1 and Zone 2. One fixed sensor covers Zone 1.	$H=\left[\begin{array}{cc} 1 & 0\\ 0.5 & 0\\ 0.5 & 0.5 \end{array}\right]\;\;\;R=\left[\begin{array}{ccc} 1 & 0 & 0\\ 0 & 1.5 & 0\\ 0 & 0 & 1.5 \end{array}\right]\;\;\;P^{+}=\left[\begin{array}{cc} 0.64 & -0.09\\ -0.09 & 0.87 \end{array}\right]$	1.511	0.550	−0.599	1.328
10	Three mobile sensors: #1 covers each available path equally, #2 covers Zone 1& Zone 2, and #3 covers both paths to Zone 1.	$H=\left[\begin{array}{cc} 0.33 & 0.33\\ 0.5 & 0.5\\ 0.5 & 0.5 \end{array}\right]\;\;\;R=\left[\begin{array}{ccc} 1.5 & 0 & 0\\ 0 & 1.5 & 0\\ 0 & 0 & 1.5 \end{array}\right]\;\;\;P^{+}=\left[\begin{array}{cc} 1.85 & -0.54\\ -0.54 & 0.87 \end{array}\right]$	2.720	1.317	0.275	1.646
